# Efficacy and Safety of Personalized Percutaneous Single-Probe Cryoablation Using Liquid Nitrogen in the Treatment of Abdominal Wall Endometriosis

**DOI:** 10.3390/jpm15080373

**Published:** 2025-08-13

**Authors:** Ghizlane Touimi Benjelloun, Malek Mokbli, Tarek Kammoun, Sinda Ghabri, Skander Sammoud, Wissem Nabi, Vincent Letouzey, Jean-Paul Beregi, Julien Frandon

**Affiliations:** 1 Department of Radiology and Medical Imaging, Nîmes University Hospital Center, University of Montpellier-Nîmes, 30900 Nîmes, France; 2 Medical Imaging Group Nîmes (MIG Nîmes), 30900 Nîmes, France; 3 Department of Gynecology and Obstetrics, Nîmes University Hospital Center, University of Montpellier-Nîmes, 30900 Nîmes, France

**Keywords:** endometriosis, abdominal wall endometriosis, cryoablation, interventional radiology, single-probe, liquid nitrogen, pain

## Abstract

**Background**: Abdominal wall endometriosis (AWE) is a rare but debilitating condition, often occurring in surgical scars after Caesarean sections. It is characterized by cyclic pain and a palpable mass, significantly impacting patients’ quality of life. Traditional treatments, including hormonal therapy and surgery, have limitations, prompting interest in minimally invasive techniques such as cryoablation. This study evaluates the efficacy and safety of percutaneous image-guided single-probe cryoablation using liquid nitrogen for symptomatic AWE. **Purpose**: To evaluate the effectiveness and safety of percutaneous image-guided single-probe cryoablation using liquid nitrogen in treating symptomatic AWE lesions, with a primary objective to assess pain relief using the Visual Analog Scale (VAS). **Materials and Methods**: This retrospective, single-center study included 14 patients (23 lesions) treated with percutaneous cryoablation between September 2022 and April 2025. Clinical, imaging (MRI and ultrasound), and procedural data were analyzed. Pain scores (VAS scale) were assessed before treatment and at 3-month follow-up. Hydro- and/or carbo-dissection were used to protect adjacent structures. Response to treatment was evaluated with MRI and clinical follow-up. Statistical analysis was performed using median, range, and percentage calculations, with comparisons made using the Mann–Whitney test. **Results**: A total of 23 AWE lesions were treated in 14 patients (mean age: 39.6 years). The median lesion volume was 3546 mm^3^, with a range from 331 mm^3^ (8 × 4.6 × 9 mm) to 45,448 mm^3^ (46 × 26 × 38 mm). Most of the lesions were located in the muscle (69.6%, *n* = 16), while 17.4% (*n* = 4) involved both muscle and subcutaneous tissue, and 13.0% (*n* = 3) were purely subcutaneous. Among the 23 treated lesions, 8.7% (*n* = 2) appeared as purely hemorrhagic, 13.0% (*n* = 3) as fibrotic, and 78.3% (*n* = 18) were classified as mixed, based on imaging characteristics. Procedures were performed under general anesthesia in 65% of cases and under sedation in 35%. Hydrodissection was used in 48% of lesions, carbo-dissection in 4%, and combined hydro–carbo-dissection in 26%. A single 13G cryoprobe was used in 83% of cases, and a 10G probe in 17%. The median ablation time was 15 min (range: 6–28 min), and the median total procedure time was 93 min (range: 22–240 min). Pain scores significantly decreased from a median of 8/10 (range: 6–10) before treatment to 0/10 (range: 0–2) at follow-up (*p* < 0.0001). MRI follow-up confirmed complete coverage of the ablation zone and disappearance of hemorrhagic inclusions in all cases. Two patients (14%) required re-treatment, both with satisfactory outcomes. No peri- or post-procedural complications were observed, and no visible scars were noted. **Conclusions**: Percutaneous cryoablation using a single probe with liquid nitrogen is a safe and effective treatment for AWE, offering significant pain relief, minimal morbidity, and excellent cosmetic outcomes. It should be considered as part of multidisciplinary care. Further prospective studies with longer follow-up are warranted to confirm these findings.

## 1. Introduction

Endometriosis is a chronic inflammatory condition that primarily affects young active women, particularly during their reproductive years, regardless of race or number of pregnancies. It is a leading cause of persistent pelvic pain, can significantly impact fertility, and adversely affects quality of life [1].

Abdominal wall endometriosis (AWE) is most commonly associated with surgical scars particularly following Caesarean sections. It is not always associated with deep pelvic endometriosis and often presents as an isolated condition. Patients typically experience cyclical symptom fluctuations, including pain and a palpable lump of the abdominal wall. Imaging techniques such as ultrasound and MRI are valuable tools for confirming the diagnosis [2,3,4,5].

Traditional treatment options for AWE to alleviate symptoms have been limited to hormonal therapies and extensive surgical interventions. However, recent studies have explored minimally invasive techniques to provide more localized symptom control. Cryoablation, in particular, has shown promise as a new treatment for AWE lesions, with favorable outcomes and low morbidity [6]. The visibility of ice formation on imaging allows for personalized treatment tailored to the size of the lesion and minimizes complications.

This single-center retrospective study aimed to assess the effectiveness and safety of percutaneous image-guided cryoablation with liquid nitrogen in treating symptomatic AWE. Our primary objective was to assess pain relief using VAS.

## 2. Materials and Methods

This observational, retrospective, monocentric non-interventional study was conducted between September 2022 and April 2025, involving 14 patients treated with single-probe percutaneous cryoablation using liquid nitrogen.

The study titled “Efficacy and Safety of Percutaneous Single Probe Cryoablation Using Liquid Nitrogen in the Treatment of Abdominal Wall Endometriosis” (ESCAWE) received a favorable opinion from the Institutional Review Board (Interface Recherche Bioéthique) of CHU Nîmes on 20 May 2025 (IRB Study Code: 25.05.01; Ref. No.: 35). The study is conducted in compliance with the General Data Protection Regulation (GDPR) and the French reference methodology MR004. All applicable ethical standards were adhered to, and an information and non-opposition note was provided in accordance with national regulations. Patients are free to refuse participation in the study after reviewing the information letter.

Patients are referred to us by their gynecologist or midwife. In some cases, the nodule is incidentally discovered on a pelvic MRI performed for the evaluation of pelvic pain. Our diagnostic radiology colleagues refer the patients to us, while also involving the hospital’s gynecologists in the care pathway, and the choice to perform cryotherapy is made in a joint decision.

Patients were primarily selected for treatment due to pain, which was often described as severe. In rare cases, although the nodule was not painful, patients requested treatment either because they could feel a palpable mass or wished to discontinue hormonal therapy. For the purpose of this study, only patients treated for painful nodules were included.

Retrospectively, relevant demographic and clinical data were collected from medical records, prior consultations, procedural reports, and post-procedure follow-up at 3 months.

All patients underwent pelvic MRI using T2-weighted sequences, T1 gradient-echo multi-echo MR sequences (Dixon), diffusion-weighted sequences, and post-contrast T1-weighted sequences. The size, location (e.g., Caesarean scar, intramuscular, subcutaneous fat, peritoneal), and number of AWE target lesions were described. The lesion type (fibrotic, hemorrhagic, or mixed) was assessed based on MRI signal characteristics and post-contrast enhancement.

All patients were seen in consultation with an expert interventional radiologist, who evaluated pain using the VAS scale. Ultrasound was used to assess the accessibility of target lesions. Personalized treatment plans and potential complications were discussed during this consultation.

Written informed consent was obtained from all patients.

The approach to personalization primarily relied on procedural adaptation—including anesthesia choice, cryoprobe gauge, dissection techniques, and image guidance methods—based on lesion shape and size, location, patient anxiety, and imaging accessibility.

The novelty lies in the use of a single-probe cryoablation system. Indeed, this system enables us to go up to 4 cm iceball size, and we could relocate the cryoprobe to treat even bigger lesions.

Procedures were performed under general anesthesia or under sedation combined with local anesthesia. We are fortunate to collaborate daily with anesthesiologists in our operating room. Anesthesia protocols may vary between practitioners, and no standardized protocol is currently in place.

Before the treatment, the lesion was identified and measured under US guidance. Probe gauge (10G or 13G) was chosen depending on the lesion size and shape (two iceball shapes are available, round and elliptic). Hydro- and/or carbo-dissection were employed to protect the adjacent organs (digestive loops, bladder, nerves) and skin if they were at risk, using Chiba needles, placed under US or CT guidance.

We then performed freeze/passive thaw/freeze/active heat cycles, with equal duration of freeze and passive thaw cycles, depending on the size of the lesions and following the manufacturer’s charts. The iceball was monitored all along the procedure under imaging guidance, and margins were assessed at the final control. Sometimes, probe relocation was needed if the nodule was too big.

Postoperatively, all patients were discharged the same day from the day hospital with well-controlled pain. A sick leave of 3 to 5 days was prescribed, along with nonsteroidal anti-inflammatory drugs (NSAIDs), level 1 analgesics, antiemetics, oral hydration, and laxatives if needed for constipation. Opioids were not required postoperatively for pain management.

Four to six weeks post-procedure, all patients underwent an MRI scan to evaluate for residual AWE lesions and potential complications. Pain and patient satisfaction were assessed during follow-up consultations at 3 months using the VAS scale and the aesthetic outcome.

Statistical analysis was performed using median, range, and percentage calculations, with comparisons made using the Mann–Whitney test. It was conducted with Microsoft Excel (Microsoft Corporation, Version 16.97.2).

This study was conducted for academic, non-profit purposes.

## 3. Results

**Figure 1:**
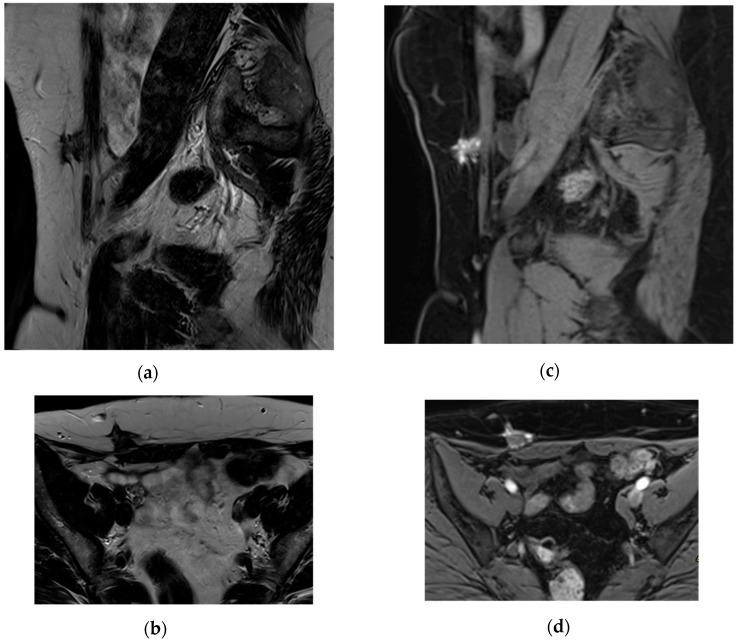
AWE on Caesarian section scar, developed in the subcutaneous fat without skin involvement, invading the underlying muscle, note the fibrous hyposignal on T2 WI (**a**,**b**) and hemorrhagic spots on T1 Water DIXON sequence (**c**,**d**).

The final sample consisted of 14 patients, with a mean age of 39.6 years, and a total of 23 ablated lesions. The median lesion volume was 3546 mm^3^, with a range from 331 mm^3^ (8 × 4.6 × 9 mm) to 45,448 mm^3^ (46 × 26 × 38 mm). Of the lesions, 69.6% (*n* = 16) were located in the muscle, 17.4% (*n* = 4) involved both muscle and subcutaneous tissue, and 13.0% (*n* = 3) were purely subcutaneous. Among the 23 treated lesions, 8.7% (*n* = 2) were purely hemorrhagic, 13.0% (*n* = 3) were fibrotic, and 78.3% (*n* = 18) were classified as mixed, based on imaging characteristics. The hemorrhagic and mixed lesions displayed “powder burn” characteristics, appearing bright on T1 fat-saturated sequences (Figure 1).

Patient characteristics and lesion details are summarized in Table 1.

The diagnosis of AWE was made based on clinical and imaging findings alone (US and MRI), which are both considered highly suggestive according to our experience and the literature. Therefore, no preoperative biopsy was performed for histological confirmation.

All patients were treated on an outpatient basis and were discharged the same day.

**Figure 2:**
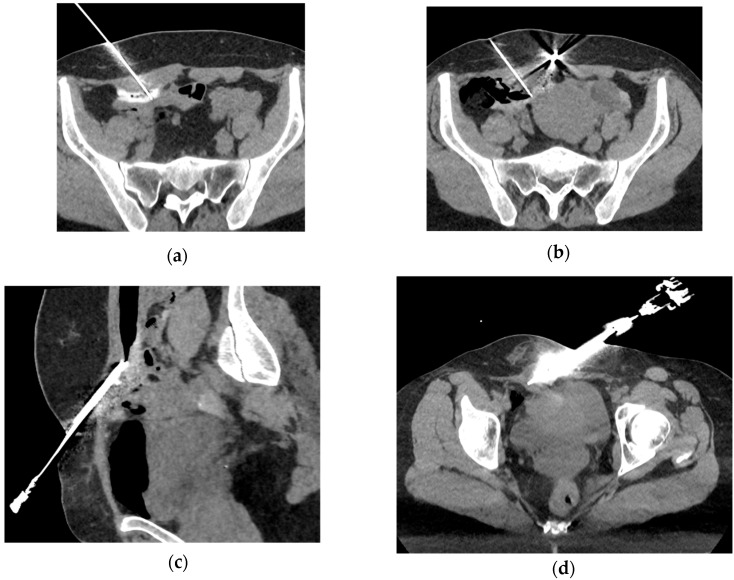
Examples of protection techniques used to prevent damaging adjacent structures (skin and bowel): hydrodissection (**a**) or carbo-dissection (**b**,**c**) are performed after Chiba needle placement between the iceball and the sensitive organs to protect the bowel, and hydrodissection of the subcutaneous fat (**d**) to protect the skin.

The treatment was performed under general anesthesia in 65% of cases (*n* = 15) and under sedation in 35% (*n* = 8). Hydrodissection was used in 48% of lesions (*n* = 11), carbo-dissection in 4% (*n* = 1), and hydro–carbo-dissection in 26% (*n* = 6) to protect the digestive loops adjacent to the abdominal wall. Additionally, hydrodissection was used in 61% of cases (*n* = 14) to protect the skin in superficial endometriotic lesions (Figure 2).

**Figure 3:**
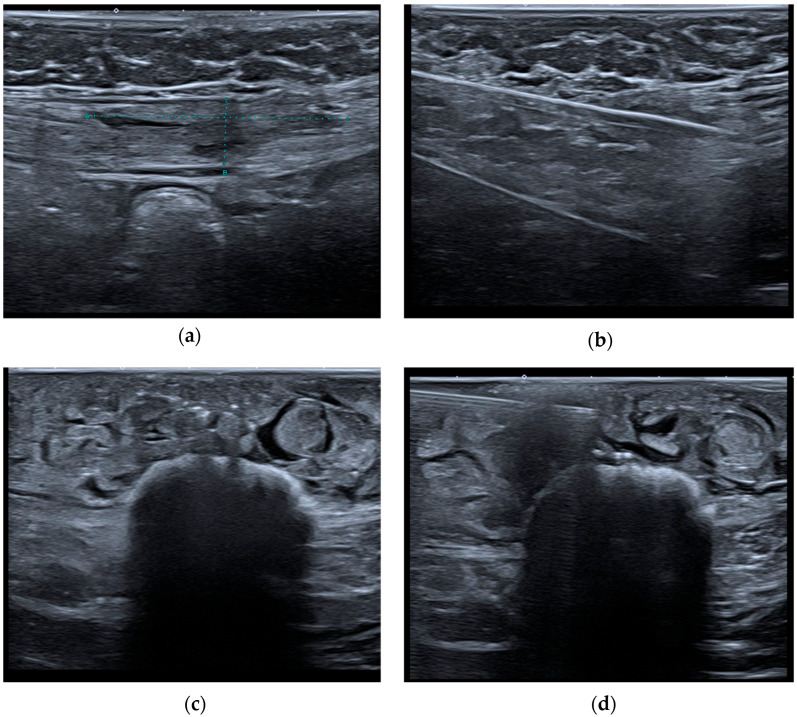
Hypoechoic nodule of the rectus abdominis muscle (**a**) corresponding to AWE lesion. Cryoablation single-probe placement (**b**) and iceball monitoring under US guidance (**c**). Chiba Needle insertion under US guidance and Hydrodissection of the skin (**d**).

A single 13G cryoprobe was used in 83% of cases (*n* = 19), and a 10G cryoprobe was used in 17% (*n* = 4). The procedure was guided by ultrasound alone in 13% of cases (*n* = 3), and by both CT and ultrasound in the remaining 87% (*n*= 20) to ensure accurate needle placement and iceball monitoring (Figure 3). Freeze–thaw–freeze cycles with active heating were applied, with a median ablation time of 15 min (range: 6–28 min), and a median total procedure time of 93 min (range: 22–240 min)

Two patients (14%) required re-treatment using the same modality. One was treated for persistent pain and a residual lesion on MRI, and the other for a new symptomatic AWE lesion occurring 13 months after the initial treatment. Both experienced satisfactory outcomes following re-treatment.

**Figure 4:**
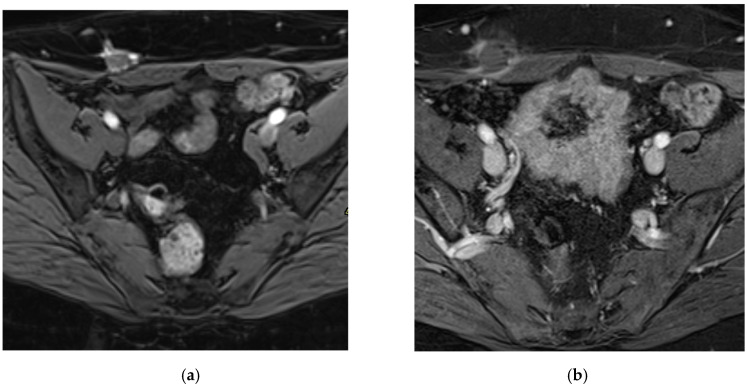
Pre- (**a**) and post-cryoablation (**b**) MRI, contrast-enhanced T1 FS WI. On post-treatment MRI, we can notice the full coverage of the AWE lesion and an inflammatory rim enhancement, corresponding to a good imaging and clinical outcome.

Pain significantly decreased from a median of 8/10 (range: 6–10) pre-procedure to 0/10 (range: 0–2) at last follow-up. A Mann–Whitney U test comparing VAS scores before and after cryoablation (*n* = 19) showed a statistically significant reduction in pain, with a *p* < 0.0001. The MRI follow-up showed accurate targeting of the ablation zone, with complete coverage of the endometriotic nodule and disappearance of the hemorrhagic inclusions (Figure 4).

## 4. Discussion

Abdominal wall endometriosis (AWE) is a rare manifestation of endometriosis, characterized by the presence of endometrial tissue within the abdominal wall, often at sites of previous surgical intervention. The incidence of AWE varies, with reports ranging from 0.03% to 3.5% among women undergoing abdominal surgeries, particularly cesarean sections [7].

Clinically, AWE typically presents as a palpable mass at or near surgical scars, accompanied by cyclic pain that correlates with the menstrual cycle. Patients often report localized discomfort or swelling in the affected area, which intensifies during menstruation. This pattern is considered pathognomonic for AWE [8].

The pathogenesis of AWE is believed to involve the direct implantation of endometrial cells into the abdominal wall during surgical procedures. These cells can proliferate and establish ectopic endometrial tissue, leading to the formation of endometriomas [7].

Given its rarity, AWE is frequently misdiagnosed, leading to significant diagnostic delays that can profoundly affect patients’ psychological well-being. On average, individuals with endometriosis experience a diagnostic delay of approximately 8.6 years, which may lead to significant psychological distress, including anxiety and depression, as patients struggle with chronic pain and the lack of a definitive diagnosis [1].

In our cohort, one patient suffered from a diagnostic delay of 3 years since the onset of symptoms.

Abdominal wall endometriosis (AWE) presents with distinct imaging characteristics across various modalities, aiding in accurate diagnosis.

Ultrasound is often the first-line imaging modality for evaluating AWE. Lesions typically appear as solid, hypoechoic masses near surgical scars, such as those from cesarean sections. These masses may exhibit irregular or spiculated margins and can show vascularity on Doppler imaging. Occasionally, cystic changes are observed within the lesions [2].

CT imaging may reveal soft-tissue masses within the abdominal wall, often located near surgical scars. These masses can have irregular borders and may infiltrate adjacent tissues. However, CT is less specific than US and MRI for characterizing soft-tissue lesions like endometriosis [9].

MRI provides superior soft-tissue contrast, facilitating detailed assessment of AWE. Typically, on T1WI, lesions often exhibit hyperintense signals due to hemorrhagic content. On T2WI, lesions may show hypointense or heterogeneous signals, reflecting varying stages of hemorrhage and fibrosis. On contrast-enhanced T1WI, AWE lesions often exhibit enhancement reflecting vascularized endometrial tissue. This enhancement is variable and may not distinctly differentiate AWE from other benign or malignant processes. On DWI, AWE lesions can show hyperintensity with corresponding hypointensity on ADC maps, indicating restricted diffusion. This restriction is attributed to the dense cellularity and fibrotic components of the lesions. The mean ADC value of AWE has been reported as 0.93 × 10^−3^ mm^2^/s. These MRI characteristics are instrumental in differentiating AWE from other soft-tissue masses [3,4,5].

In our cohort, the imaging findings (US and MRI) matched the semiological features described in the literature.

In summary, integrating clinical history with imaging findings—such as hypoechoic masses on US and hyperintense signals on T1WI MRI—enhances the diagnostic accuracy for AWE. Thus, no pre-procedural biopsy was needed to confirm the diagnosis of AWE lesions in our patient cohort.

Cryoablation offers notable advantages over other ablative techniques in treating soft-tissue masses, including abdominal wall endometriosis (AWE), particularly concerning pain mitigation and aesthetic outcomes.

Indeed, cryoablation allows for precise visualization of the ablation zone. The iceball formed during the procedure is clearly delineated on imaging modalities such as ultrasound, CT, and MRI. This real-time monitoring facilitates accurate targeting and preservation of surrounding healthy tissues [10].

Furthermore, the cooling effect of cryoablation exerts an anesthetic property, leading to less procedural pain compared to heat-based methods like radiofrequency ablation (RFA) or microwave ablation (MWA). This often enables the use of local anesthesia and moderate sedation instead of general anesthesia [11]. In our cohort, both general anesthesia and sedation were used. The choice is adapted to each situation and depends on the evaluation of the radiologist, considering multiple parameters as the number and size of the lesions, the anxiety of the patient, and the complexity of the procedure.

Furthermore, Cornelis et al. reported that cryoablation led to substantial pain reduction and tumor size decrease in soft-tissue tumors, with a low incidence of severe adverse events [12]. In our study, pain decreased significantly from a median of 8/10 (range: 6–10) pre-procedure to 0/10 (range: 0–2) at last follow-up with *p* < 0.0001, and no peri- or post-operative adverse events were observed. These data confirm the efficacy and the safety of this technique.

Enhanced aesthetic outcomes are another advantage of percutaneous cryoablation. By minimizing damage to adjacent tissues and reducing inflammatory responses, cryoablation is associated with superior cosmetic results. This is particularly beneficial in treating superficial lesions where aesthetic considerations are paramount in a young female population [12,13]. In our patient cohort, no visible scar was observed.

One disadvantage of cryoablation is its longer procedural duration compared to microwave ablation (MWA) and radiofrequency ablation (RFA), as it requires at least two freeze cycles and one thaw cycle to achieve effective tissue destruction. This prolonged treatment time may be a consideration in clinical decision-making, particularly for larger lesions [14]. However, the small size of AWE lesions and the use of a single-probe technique minimize the impact of this longer treatment time. The median total procedure time in our cohort was 93 min, including patient setup and anesthesia time, which seems to be a reasonable duration.

Considering the cost-effectiveness of cryoablation as an alternative to surgical excision for treating AWE, although the initial expense of cryoprobes is relatively high, the overall cost of cryoablation can be lower than that of conventional surgery, depending on the country and the structure. A study comparing percutaneous cryoablation to surgery for extra-abdominal desmoid tumors—a condition with similarities to AWE—found that cryoablation resulted in cost savings, primarily due to reduced hospital stays and lower complication rates [15].

Single-probe cryoablation using liquid nitrogen adds another advantage to the technique compared to other cryoablation devices, as it is more cost-effective than those using Argon or Helium gas due to the large expense associated with their purchase and storage [14,16].

In the context of AWE, cryoablation has demonstrated similar effectiveness to surgery, with additional benefits such as shorter hospitalization and fewer complications. A study reported that patients undergoing cryoablation had a median hospital stay of 0.8 days compared to 2.8 days for those who underwent surgery. Additionally, 23.1% of surgical patients (3 out of 13 patients) experienced severe complications, whereas no severe complications were observed in the cryoablation group [12]. However, larger surgical studies report lower complication rates [17]. In our study, all patients were treated on an outpatient basis.

These factors contribute to the cost-effectiveness of cryoablation, offsetting the higher initial cost of cryoprobes and making it a financially viable option for AWE treatment.

In summary, cryotherapy treatment of parietal endometriosis lesions is planned in a personalized manner for each patient preoperatively, based on imaging characteristics (number, size, location, adjacent structures at risk) and the patient’s profile. The final outcome is scar-free with significant clinical improvement, particularly regarding painful symptoms. These results confirm what has been reported in the literature about interventional radiology treatment of parietal endometriosis and reinforce the idea of moving toward less invasive, more precise medicine tailored to each situation.

Table 2 summarizes the findings of our study and the results of other published studies on cryoablation and/or surgery for AWE.

This study, however, has several limitations that must be acknowledged. First, it is a retrospective, single-center study with a small sample size (14 patients, 23 lesions), which limits the statistical power and generalizability of the findings. The absence of a control group (e.g., surgical or medical management) prevents any direct comparison of cryoablation with standard treatment options.

Second, the follow-up period was limited to 3 months, which is insufficient to assess the long-term efficacy of cryoablation, recurrence rates, or durability of pain relief in a chronic and often recurrent condition such as endometriosis. This is an additional limitation of the retrospective study. In practice, patients return to their gynecologist for follow-up. In the event of symptom recurrence, they either contact us directly or are referred back by their gynecologist. A longer-term follow-up organized within interventional radiology is necessary to determine sustained outcomes and potential delayed complications.

Third, pain assessment relied exclusively on the VAS scale, which, while practical, remains subjective and susceptible to patient interpretation and reporting variability. No standardized quality-of-life questionnaires or functional outcomes were included.

These limitations highlight the need for prospective, multicenter studies with larger cohorts, standardized protocols, and longer follow-up to confirm the safety and effectiveness of cryoablation in the management of AWE.

## 5. Conclusions

In conclusion, percutaneous single-probe cryoablation using liquid nitrogen is an effective and safe treatment for AWE, particularly for younger patients, offering low post-operative morbidity and minimal adverse events. It is a viable alternative to traditional surgery, which typically involves general anesthesia, large surgical excisions, and considerable aesthetic damage.

Treatment decisions should be discussed in a multidisciplinary setting involving radiologists, interventional radiologists, and gynecologists. Future work should aim to define a more robust framework for personalized treatment of AWE, including clinical profiles, imaging features, and potentially biomarkers.

A 3-month follow-up is clearly insufficient to determine sustained outcomes in a chronic and recurrent condition such as endometriosis. Further cross-sectional studies and long-term follow-up organized within interventional radiology are necessary to fully assess the long-term results of this modality.

## Figures and Tables

**Table 1:** Patient characteristics and lesion details.

Number of Patients	14
Total Number of Lesions	23
Median Age	39 years
Median Lesion Volume and Range	3546 mm^3^, with a range from 331 mm^3^ (8 × 4.6 × 9 mm) to 45,448 mm^3^ (46 × 26 × 38 mm).
Imaging Characteristics	- Mixed (*n* = 18) - Hemorrhagic (*n* = 2) - Fibrotic (*n* = 3)
Lesion Locations	- Rectus abdominis muscle (*n* = 16) - Subcutaneous fat (*n* = 3) - Combined rectus abdominis + subcutaneous fat (*n* = 4)

**Table 2:** Comparison between our study findings and other published studies on cryoablation or surgery for AWE.

Study	Treatment Modality	Patients (*n*)	Median Follow-Up	Symptom-Free Rate at FU	Pain Reduction (VAS)	Aesthetic Outcome	Complications	Hospital Stay	Recurrence Rate
Touimi Benjelloun et al., 2025	Cryoablation (single probe, liquid N2)	14 (23 lesions)	3 months	86% (12/14)	8→0	No visible scar	None	Outpatient	14% (2/14)
Maillot et al., 2017 [12]	Cryoablation vs. Surgery	7 cryoablation/13 surgery	22.5 months for cryoablation/54 months for surgery	66.7% cryoablation/92% surgery	NR	No visible scan: cryoablation/69% aesthetic sequels: surgery	0% cryoablation/ 23% surgery (severe complications)	0.8 d cryoablation/2.8 d (surgery)	14% cryoablation/7.7% surgery
Dibble et al., 2017 [11]	Cryoablation	3	2.5 months	2/3 patients	8→0	NR	None	Outpatient	NR
Najdawi et al., 2023 [18]	Cryoablation	42	13.5 months	82.72%	8→0	NR	9.5% mild adverse events (skin burn); 2% severe adverse event (skin burn and small bowel injury)	Outpatient	9.5%
Hasan et al., 2021 [19]	Surgical excision	30	38 months (mean)	100% (no recurrence)	VAS NR	NR	33% (1 hernia, 5 wound infections, 4 bleedings)	Not reported	0%
Horton et al., 2008 [20]	Surgical excision	445 (445 lesions)	NR	95% (symptom relief)	VAS NR	NR	NR	Not reported	4.3% (Range 0% to 29%)
Benedetto et al., 2022 [17]	Surgical excision	83	US 6 mo after surgery + annual US	NR	Pre-op: 85% VAS ≤ 6, 15% ≥ 7; post-op: NR	NR	Hematoma/seroma 10.8%; Incisional Hernia 2.4%; (mostly > 30 mm lesions); no major events	3 to 36 h (mean 16 h)	0%

## Data Availability

Dataset available on request from the authors.
